# Recurrent pneumonias and bronchiectasis associated a commom variable immunodeficiency

**DOI:** 10.1186/1939-4551-8-S1-A275

**Published:** 2015-04-08

**Authors:** Thais Martins Souza

**Affiliations:** 1 Ufjf, Brazil

## Introduction

Commom variable immunodeficiency (CVI) is the second more comom cause of Immunodeficiency. Its prevalence is about 10 000 a 50 000 persons. Its incidente is about the first and second life's decade. Both genders are commited and it can be associated a several phenotypes such a recurrent inlfections,autoimmunity,enteropathy,neoplasms,polyclonal lymphocytic infiltration. The main recurrent infections are those that achieve lungs,paranasal sinuses and gastrointestinal . 60% of the cases of CVI are associated a bronchiectasis. The diagnosis is based on current infections ,reduction of IGG , igm and /or iga below 2 standarts desviations for age , age over 4 years old and excluding others causes of hypogammaglobulinemia.

## Objective

The objective is to report the case of a patient who was diagnosed with CVI associated a recurrent pulmonary infections and bronchiectasis.

## Description of the case

GOA,16 years old, 6 previous pneumonias . The first one was when she was 6. She was hospitalized In two of those episodes and also showed weight loss .when she was 15, the investigation was started. Sweat test and anti HIV were negative.low levels of Immunoglobulins were found (IgG= 20,1 mg/dl; IgM=32,2 mg/dl; IgA=7,2 mg/dl; IgE=1,1 mg/dl). The lymphocite immunophenotyping search was normal for her age (CD3=2939mm^3^, CD4=693mm^3^, CD8=2011mm^3^, CD19=876mm^3^). Tomografy of the chest presented suggestive areas of severe chronic bronchiectasis. After the exclusion of others hypogammaglobulinemias ' causes and the diagnostic of CVI being confirmed, the treatment was iniciated using intravenous imunoglobulin 400 mg/kg/day and antimicrobial prophylaxis with azitrhomicin. After initiation of treatment, the child didn t have others infections and Gained satisfactory weight. Currently she is being monitorized In a immunology ambulatory.

## Conclusion

We want to emphasize need to exclude CVID In patients that presents recurrent pneumonias, mainly when it’s associated a bronchiectasis.

## Consent

Written informed consent was obtained from the patient for publication of this abstract and any accompanying images. A copy of the written consent is available for review by the Editor of this journal.
